# Suprainguinal Lateral Femoral Cutaneous Neurectomy for Recurrent Meralgia Paresthetica: A Technique Guide

**DOI:** 10.1002/atn2.70013

**Published:** 2026-04-29

**Authors:** Joshua T. Finerty, Allison R. Garden, Manuel A. Romero‐Padron, Joshua S. Everhart

**Affiliations:** ^1^ Department of Orthopaedic Surgery Indiana University School of Medicine Indianapolis Indiana U.S.A.; ^2^ University of Kentucky College of Medicine Lexington Kentucky U.S.A.; ^3^ Edward Via College of Osteopathic Medicine‐ Carolinas Spartanburg South Carolina U.S.A.

## Abstract

Meralgia paresthetica (MP) is a neuropathy caused by injury or compression of the lateral femoral cutaneous nerve (LFCN), presenting as numbness, tingling, or burning pain in the lateral thigh. While surgical decompression has traditionally been a common surgical treatment for MP, recently, fully transecting the LFCN is increasingly becoming the first‐line surgical management as more favorable outcomes are reported. Although infrainguinal neurectomy is the primary surgical approach, recurrent or persistent symptoms due to neuroma formation or proximal variations in branching patterns can necessitate revision surgery via a suprainguinal approach. This technical note details our preferred suprainguinal LFCN neurectomy technique for the treatment of recurrent MP.

## Graphical abstract

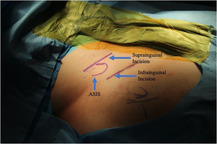

**VIDEO 1:** Patient was placed supine on the operating table. The right hip was prepped and draped in sterile fashion. An oblique incision was made along the inguinal crease several centimeters distal to the anterior superior iliac spine near previous incision as this was an example of a revision surgery. Dissection through this scar tissue was performed. A neuroma is present at the distal end of the lateral femoral cutaneous nerve (LFCN) and excised. Then, the LFCN trunk was tagged with polypropylene (Prolene) suture just distal to the inguinal ligament. Next, attention was turned to the suprainguinal approach and an oblique incision several centimeters proximal to the ASIS was made. Using a retroperitoneal approach, the LFCN within the pelvis on the anterior surface of the iliacus muscle was exposed. The tagged nerve end underneath the inguinal ligament was retrieved to confirm that the correct nerve was identified.The LFCN was transected approximately 5 centimeters proximal to the inguinal ligament. Video content can be viewed at https://doi.org/10.1002/atn2.70013.

The lateral femoral cutaneous nerve (LFCN), originating from the L2 and L3 nerve roots, innervates the skin over the anterolateral thigh. Meralgia paresthetica (MP) results from compression or trauma to this nerve.^1^ The LFCN originates at the lateral border of the psoas major muscle, courses obliquely across the surface of the iliacus muscle toward the anterior superior iliac spine (ASIS), and, in the majority of cases, exits the pelvis beneath the inguinal ligament. Although anatomical variations occur, the nerve typically passes medial to, or directly through, the sartorius muscle as it enters the anterior thigh.^2^

A population‐based study reported an incidence rate of approximately 32.6 per 100,000 patient‐years, noting higher prevalence with advanced age, obesity, and diabetes mellitus.^3^ MP is additionally recognized as a complication following open anterior approaches to the hip as well as hip arthroscopy, as the typical trajectory of the LFCN near the ASIS places it at risk during standard anterior portal placement.^4^ Surgical management is considered following failed conservative therapy, and options typically involve infrainguinal neurectomy or decompression.^5^ However, when symptoms persist or recur after primary neurectomy, re‐resection is considered. The suprainguinal approach facilitates proximal nerve transection above the inguinal ligament, aiming to minimize symptomatic recurrence by reducing neuroma formation and mechanical irritation at the site of previous transection. This approach also minimizes the likelihood of persistent symptoms due to uncommon proximal variations in nerve branching, including branching at or proximal to the inguinal ligament. This technical note outlines the surgical technique for suprainguinal LFCN neurectomy (Video 1).

### PATIENT EVALUATION, IMAGING, AND INDICATIONS

Initial evaluation of MP begins with a thorough history and physical examination. Patients typically report anterolateral thigh discomfort described as burning, tingling, or hypersensitivity to light touch or clothing.^6^ Physical examination maneuvers include reproduction of symptoms with localized pressure or percussion just inferior to the ASIS, known as Tinel's sign.^7^ A diagnostic injection of local anesthetic in this region can provide temporary relief and serve as a confirmatory test.^8^ In our experience, diagnostic relief of symptoms of only a portion of the affected region of the thigh with an infrainguinal LFCN local anesthetic block is suggestive of proximal branching variations of the LFCN. In this scenario, a suprainguinal LFCN approach should be considered to ensure that the neurectomy is proximal to the site of nerve branching. Imaging studies such as ultrasonography or magnetic resonance imaging may be used to evaluate for alternative diagnoses or to assess the course of the nerve, particularly in cases involving suspected mass effect or variant anatomy.

In patients presenting with recurrent or persistent symptoms following previous infrainguinal LFCN neurectomy or decompression, evaluation should confirm that the original diagnosis of MP remains accurate. Symptom localization should still correlate with the LFCN distribution, and a diagnostic nerve block proximal to the previous surgical site may again help to confirm the diagnosis. Imaging, particularly high‐resolution ultrasonography or magnetic resonance imaging, may be used to assess for neuroma formation, scarring, or atypical proximal nerve branching that may have been missed during the initial surgery. A history of temporary symptom relief following the primary neurectomy, followed by recurrence after a pain‐free interval, is suggestive of symptomatic neuroma and often warrants consideration of revision neurectomy through a suprainguinal approach.

## SURGICAL TECHNIQUE

**FIGURE 1:**
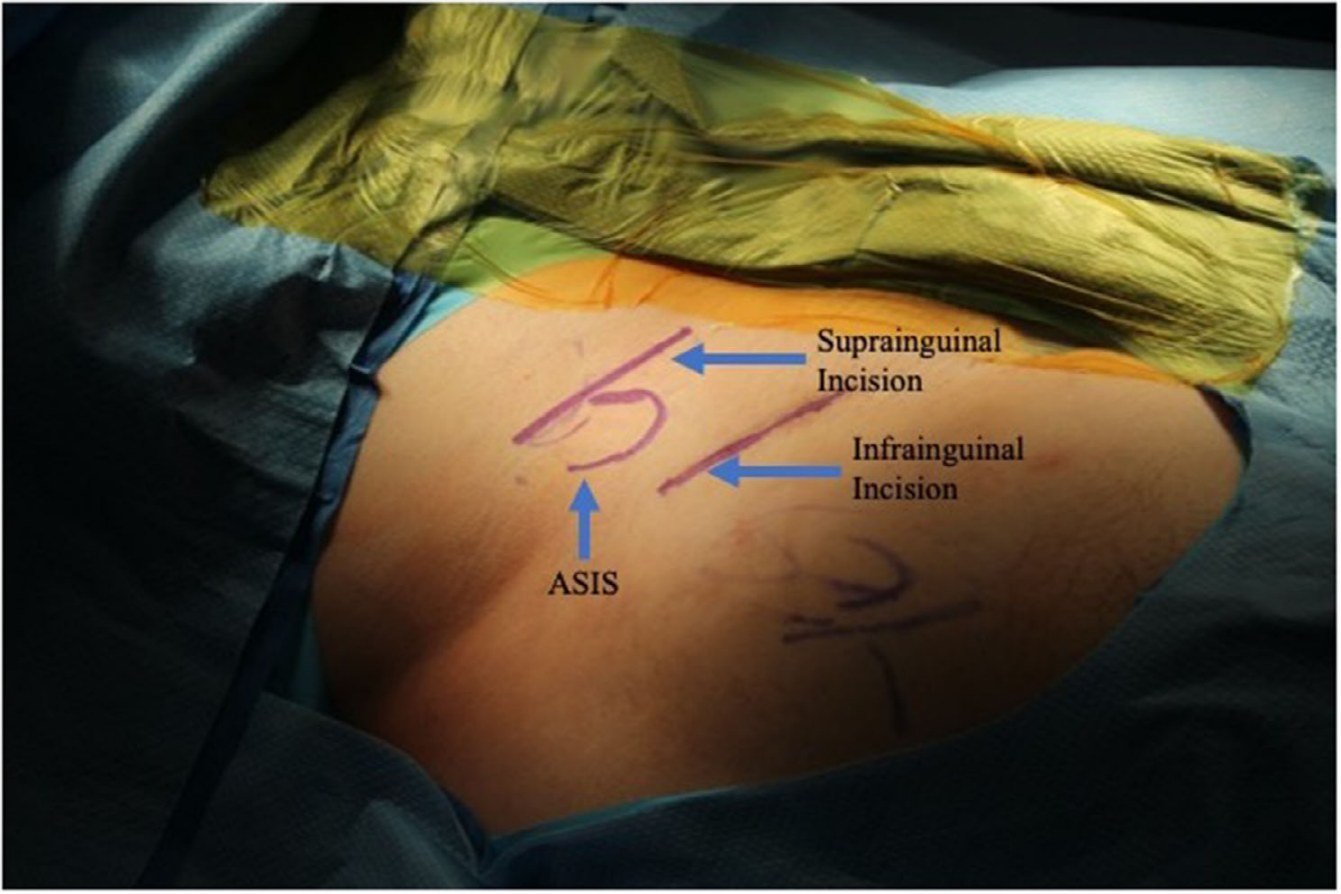
Image of patient positioning. Patient is supine on the operating table. Right anterolateral thigh is prepared in standard fashion. The anterior superior iliac spine (ASIS) is outlined in curved purple marker (signaled by arrow). Suprainguinal and infrainguinal incisions to be made on the straight purple lines (see arrows).

The patient is placed supine on the operating table. The lower abdomen and proximal lateral thigh, with wide exposure around the ASIS, should be prepped in a sterile fashion (Figure 1).

### Infrainguinal Neurolysis and Neuroma Excision

**FIGURE 2:**
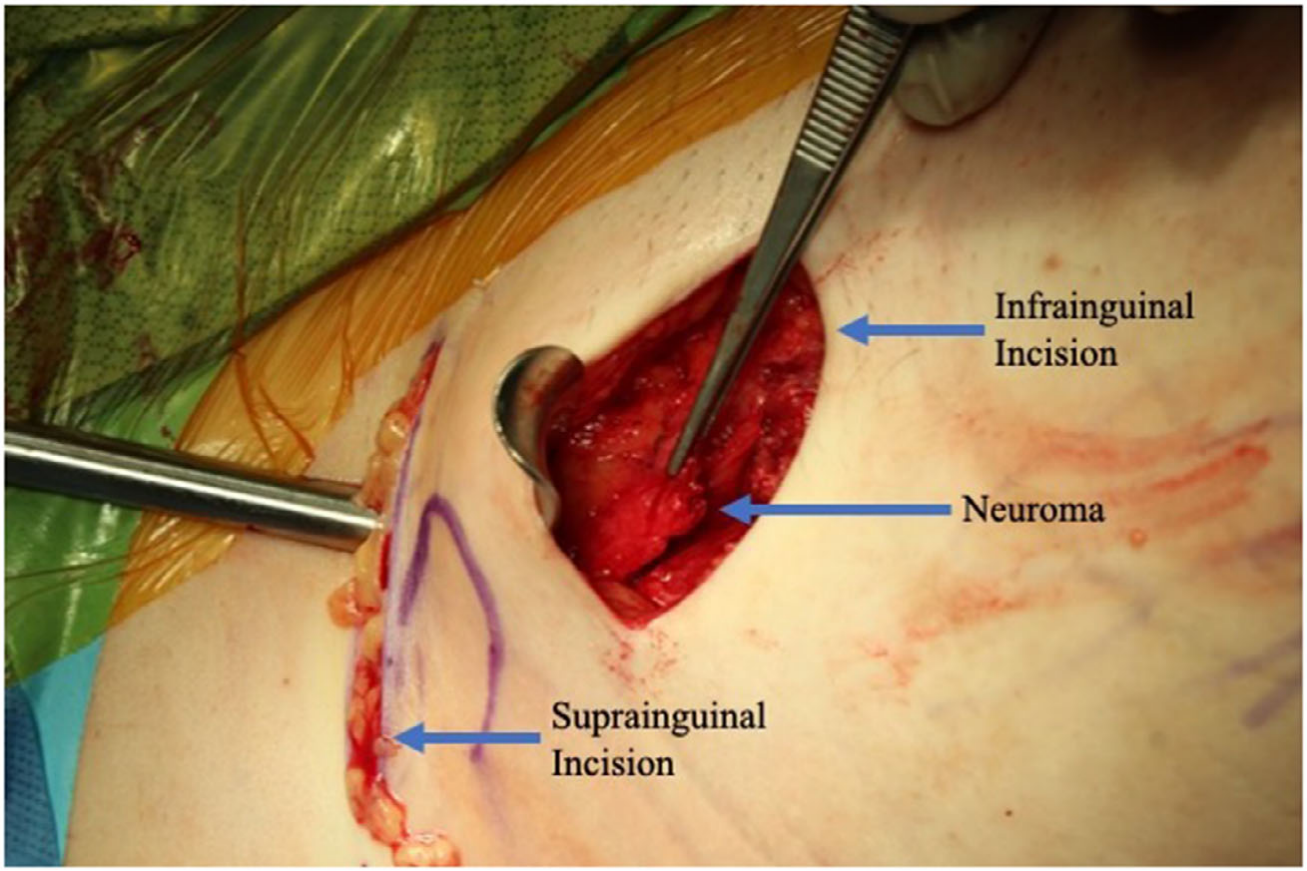
Right hip, supine position. View of the neuroma prior to the neurectomy via the infrainguinal incision. Neuroma, infrainguinal incision, and suprainguinal incision are signaled by arrows.

In the case of revision LFCN neurectomy after a prior infrainguinal neurectomy or decompression, an oblique incision should be made several centimeters distal to the ASIS. Subcutaneous flaps should be developed, and the fascia overlying the sartorius, inguinal ligament, and tensor fascia lata should be easily visualized. There is typically a consolidation of the fascia with underlying fibrous scar tissue in the case of prior surgery at this site. Neuroma tissue is typically confluent with fibrous tissue in the fascial layers, and careful dissection is required to isolate the neuroma. For this reason, at the time of primary infrainguinal neurectomy, we prefer to use blue‐dyed nonabsorbable polypropylene suture for the nerve end implantation into muscle to facilitate identification of the neuroma in the event that revision surgery is required.^9^ If a neuroma is present at the distal end of the LFCN, excise the neuroma (Figure 2). Then, tag the LFCN trunk with a suture just distal to the inguinal ligament. This step will help facilitate identification of the LFCN nerve trunk during the subsequent suprainguinal approach.

### Suprainguinal Neurectomy and Nerve End Implantation

**FIGURE 3:**
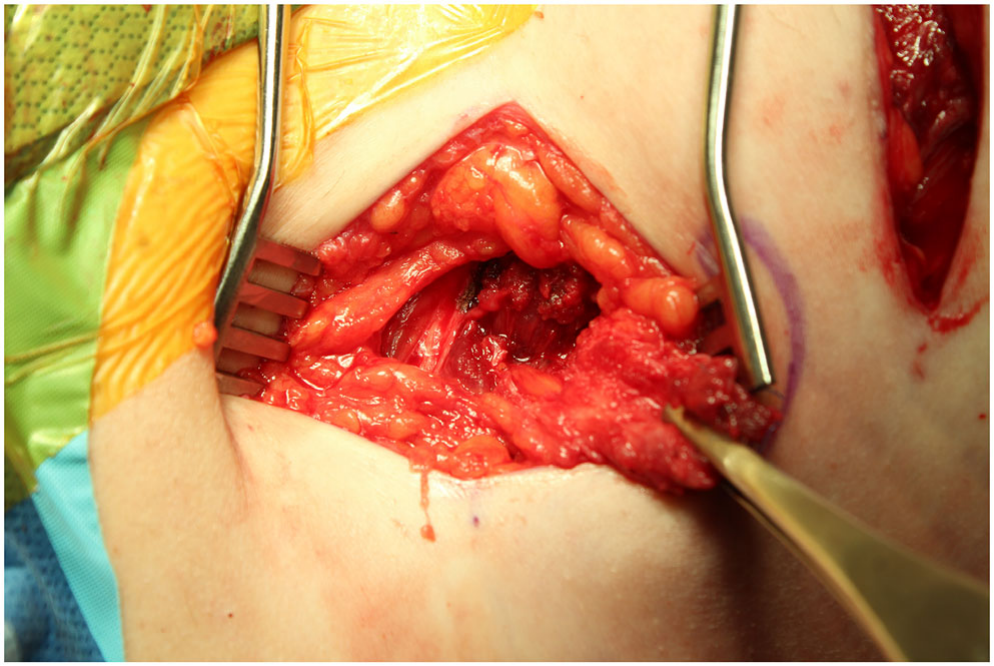
Right hip, supine position. View of the process of dissection via the suprainguinal approach. The neuroma tissue that was pulled proximally underneath the inguinal ligament is reflected in this image with forceps.

**FIGURE 4:**
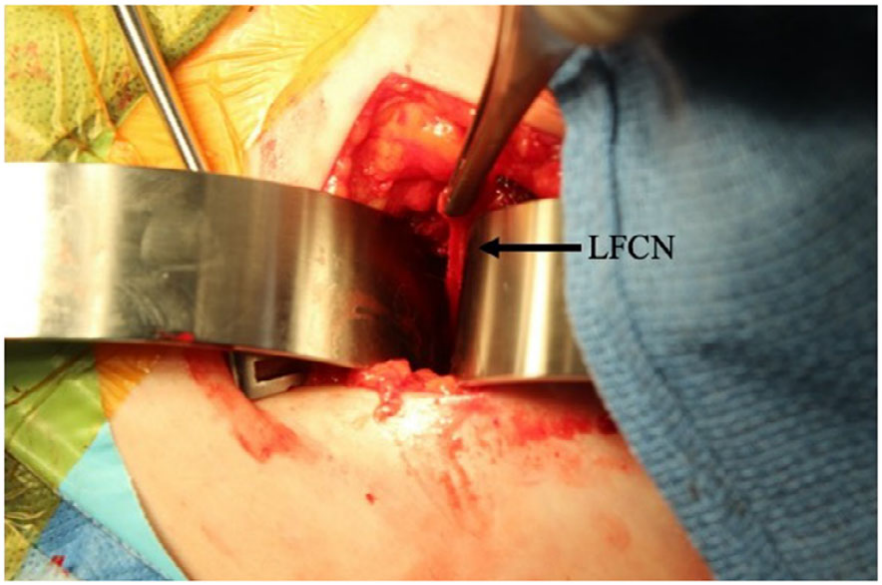
Right hip, supine position. View of the lateral femoral cutaneous nerve (LFCN) after dissecting surrounding tissue via the suprainguinal incision. In this image, the LFCN trunk is held by forceps and signaled by an arrow.

A suprainguinal oblique incision is made several centimeters proximal to the ASIS and parallel to the iliac crest (Figures 1 and 2). The abdominal wall musculature from the ASIS and anterior iliac crest is partially detached, with a cuff of tissue left for later end‐to‐end repair. Using a retroperitoneal approach, the LFCN trunk is exposed within the pelvis on the anterior surface of the iliacus muscle (Figure 3). The previously tagged distal nerve end underneath the inguinal ligament is retrieved to confirm that the LFCN was correctly identified within the pelvis. Utilization of anatomic landmarks is very important at this step, particularly if a suprainguinal approach is being performed as a primary procedure for meralgia paresthetica, as there have been reports of identification of the incorrect nerve even by an experienced surgeon with the suprainguinal approach to the LFCN.^10^ The LFCN trunk is then transected as proximal as possible; in practice, we perform the nerve transection approximately 5 cm proximal to the inguinal ligament (Figure 4). The anterior surface of the iliacus muscle can be utilized for nerve end implantation, which we typically perform with dyed, permanent 4‐0 polypropylene suture. One of the proposed purposes of this step is to help shield the nerve end from external mechanical stimulation, potentially reducing the severity of neuroma‐related symptoms.^11^ While this technique does not eliminate the risk of neuroma formation, it is associated with more organized nerve regeneration and typically results in smaller, less symptomatic neuromas. It is unclear whether this step is as important for a suprainguinal neurectomy as an infrainguinal neurectomy, because the intrapelvic portion of the LFCN should not be exposed to as much mechanical stress or compression as the LFCN at or distal to the inguinal ligament. The abdominal musculature should be repaired end‐to‐end to the cuff of tissue left on the anterior iliac crest and ASIS, followed by a superficial layered closure.

### Postoperative Care and Recovery

Acute nerve pain flares or temporary phantom pain may occur following neurectomy. We recommend an oral corticosteroid taper to be taken as needed to manage these episodes. We routinely prescribe a skeletal muscle relaxant and nonsteroidal anti‐inflammatory medication in addition to a 3‐day supply of oral opioids to be taken as needed. Although in our experience, wound healing complications are lower with the suprainguinal approach compared with incisions at or below the inguinal crease in patients with obesity, patients should be advised to promptly notify the provider of any early signs of infection. We do not routinely use a surgical drain for this procedure. To minimize the risk of seroma formation, patients are advised to avoid prolonged walking or strenuous physical activity for 2 to 3 weeks after surgery. Additionally, patients are advised to minimize core muscle activation during the first 4 weeks after surgery, as a small portion of the anterior abdominal wall musculature was taken down and subsequently repaired.

## DISCUSSION

One major advantage of the suprainguinal approach for lateral femoral cutaneous neurectomy is that transection occurs significantly proximal to the inguinal ligament. This technique minimizes the risk of symptomatic neuroma occurrence by preventing mechanical irritation and tethering of the nerve stump, which is common at or below the inguinal ligament.^11^, ^12^ Additionally, because the nerve is transected well proximal to the inguinal ligament, this approach may be especially beneficial for patients with atypical nerve branching patterns at or proximal to the inguinal ligament or those whose LFCN symptoms are exacerbated by subcutaneous abdominal fat or prolonged nerve compression.

The suprainguinal approach allows direct visualization of the LFCN at the proximal level, providing better management of anatomical variants or unexpected branching patterns. Others have highlighted that unusual anatomical configurations (e.g., bifurcation or proximally located branches) may be inadequately addressed via infrainguinal methods.^10^ A proximal, suprainguinal dissection is particularly advantageous for identifying and addressing these variations, improving the likelihood of complete symptom relief.

The suprainguinal approach allows for a retroperitoneal trajectory, which enables direct visualization of the anterior aspect of the iliacus muscle. This expanded exposure facilitates identification of proximal nerve branches that may be inaccessible through infrainguinal dissection. By accessing the LFCN as it courses over the iliacus, the surgeon gains better control over proximal nerve trunk identification and resection.

In revision surgeries, re‐exploring the original infrainguinal incision allows for distal identification of the LFCN. The nerve can then be traced proximally into the pelvis, serving as a safeguard to confirm correct nerve isolation. This step is particularly valuable in minimizing the risk of misidentifying adjacent sensory nerves, such as the iliohypogastric or ilioinguinal nerves. There has also been a report of incorrect identification of the femoral nerve as the LFCN with this approach.^10^ Tracing the tagged nerve proximally enhances procedural accuracy and reduces the likelihood of inadvertent neurectomy of nontarget structures.

It is important to consider several technical pearls when performing the suprainguinal approach for LFCN neurectomy (Table 1). Key considerations include the retroperitoneal anatomy of the nerve on the anterior iliacus surface, the need to detach abdominal musculature from the ASIS for adequate exposure, and the value of using nonabsorbable, dyed polypropylene suture to mark the nerve in case revision is required. In revision cases, tracing the nerve proximally from a prior infrainguinal incision can help confirm identification and minimize the risk of injuring nearby sensory nerves such as the ilioinguinal or iliohypogastric nerves. The advantages of this technique include improved nerve visualization, the ability to transect the LFCN far proximal to the inguinal ligament to reduce neuroma formation, and the simplicity of muscle implantation to reduce painful nerve regrowth (Table 2). As with any surgical procedure, there are risks, including the potential for symptomatic neuroma formation and the need for meticulous incision care, especially in patients with obesity. This technique has proven to be reliable in our experience, offering a safe and effective option for patients with recurrent or persistent meralgia paresthetica following prior infrainguinal LFCN neurectomy.

**TABLE 1:** Surgical Pearls for Suprainguinal Approach for LFCN Neurectomy

1. The LFCN trunk is easier to identify proximal to inguinal ligament in the case of uncommon LFCN anatomic variants (branching proximal, through, or anterior to the inguinal ligament)
2. The LFCN lies on anterior surface of iliacus muscle and is extraperitoneal
3. Must detach abdominal wall musculature from the anterior iliac crest and ASIS to access LFCN proximal to inguinal ligament. A cuff of tissue should be left on the iliac crest for later end‐to‐end repair of the abdominal wall.
4. Utlization of dyed, nonabsorbable suture such as polypropylene (Prolene) for nerve end implantation into muscle can help tidentify the desired nerve in the case of neuroma formation in the event that a revision is required
5. If anatomy is in doubt, consider use of an intraoperative nerve stimulator to confirm that the isolated nerve is not the femoral nerve. Inadvertent isolation of the femoral nerve has been reported with suprainguinal approach.^10^

ASIS, anterior superior iliac spine; LFCN, lateral femoral cutaneous nerve.

**TABLE 2:** Advantages, Risks, and Limitations for Suprainguinal Approach for LFCN Neurectomy

**Advantages**	**Risk, Limitations, and Pitfalls**
‐ The LFCN can be transected far proximal to inguinal ligament, therefore minimizing risk of symptomatic neuroma ‐ Simple approach ‐ Ease of identification of the LFCN ‐ Nerve implantation into muscle reduces the risk of phantom pain or painful neuroma	‐ Can develop symptomatic neuroma ‐ Care must be taken to keep incision clean to avoid infection, particularly in patients with obesity ‐ Care must be taken to avoid mistaking the LFCN for nearby sensory nerves (iliohypogastric, ilioinguinal, etc.) or the femoral nerve.

LFCN, lateral femoral cutaneous nerve.

## DISCLOSURES

The authors (J.T.F., A.R.G., M.A.R‐P., J.S.E.) declare that they have no known competing financial interests or personal relationships that could have appeared to influence the work reported in this paper.
